# Genome-wide association study identifies key F-box genes linked to ethylene responsiveness and root growth in rice (*Oryza sativa* L.)

**DOI:** 10.3389/fpls.2024.1501533

**Published:** 2024-12-18

**Authors:** Suparad Klinsawang, Wanchana Aesomnuk, Piyamongkol Mangkalasane, Vinitchan Ruanjaichon, Jonaliza L. Siangliw, Bipin K. Pandey, Malcolm J. Bennett, Samart Wanchana, Siwaret Arikit

**Affiliations:** ^1^ Department of Agronomy, Faculty of Agriculture at Kamphaeng Saen, Kasetsart University, Nakhon Pathom, Thailand; ^2^ Rice Science Center, Kasetsart University, Nakhon Pathom, Thailand; ^3^ National Center for Genetic Engineering and Biotechnology (BIOTEC), National Science and Technology Development Agency (NSTDA), Pathum Thani, Thailand; ^4^ School of Biosciences, University of Nottingham, Leicestershire, United Kingdom

**Keywords:** rice, root, ethylene sensitivity, GWAS, F-box

## Abstract

Rice (*Oryza sativa* L.) is a staple food for more than half of the world’s population, but its yields are increasingly threatened by environmental problems, including soil compaction. This problem limits root growth which limits water and nutrient foraging capacity thus reduces productivity due to, restricted diffusion of ethylene, a key plant hormone playing an important role in exacerbating these effects. Elevated ethylene levels in compacted soils can further inhibit root development. However, rice varieties that are less sensitive to ethylene may have an advantage as they exhibit better root growth and resource utilization under such conditions. In this study, 220 diverse rice accessions were analyzed to uncover the genetic factors that influence root length reduction (RLR) in response to ethylene. Genome-wide association studies (GWAS) identified a significant QTL on chromosome 10, named *qRLR10*, associated with ethylene response. Within this region, 20 candidate genes were identified, with three F-box genes namely *Os10g0124700*, *Os10g0126600* and *Os10g0128200* showing a strong correlation with RLR variations. These genes are involved in protein degradation, root development and hormone signaling, indicating their possible role in regulating ethylene sensitivity. The results suggest that rice varieties with lower ethylene sensitivity may have better root growth in compacted soils, making them ideal targets for breeding programs aimed at improving resilience to harsh environmental conditions. These results underscore the critical role of ethylene in rice root development and provide valuable insights for future rice improvement strategies aimed at mitigating the effects of soil compaction.

## 1 Introduction

Rice (*Oryza sativa* L.) is one of the most important staple crops that feeds more than half of the world’s population. Thailand, a major player in global rice production, ranks as the sixth-largest rice producer, following China, India, Indonesia, Bangladesh, and Vietnam (FAO, 2017). Farming practices in Thailand and other countries have significantly evolved over the years, with mechanization becoming increasingly central. The use of heavy machinery, such as power tillers, tractors, and harvesters, has become widespread in agricultural operations, including soil preparation, sowing, and harvesting (FAO, 2017). While these advancements have boosted agricultural productivity, they have also led to unintended consequences, particularly soil compaction, which poses a significant challenge to crop growth and yield (Hassan et al., 2007). Soil compaction, a condition where soil particles are pressed together, reducing macropore spaces, is a major constraint in modern agriculture. It negatively impacts crop production by restricting root growth, limiting soil water and nutrient availability, and decreasing soil porosity (Arocena, 2000; Khan et al., 2012). Additionally, soil compaction hinders gas exchange between plant roots and the surrounding environment, further exacerbating plant stress. In this context, plants naturally produce various volatile phytohormones, such as ethylene, which can diffuse through air spaces between soil aggregates in noncompacted soil. However, soil compaction restricts ethylene diffusion in soil micropores, leading to inhibited root elongation and reduced overall root growth, which negatively impacts the plant’s ability to access water and nutrients (Pandey et al., 2021; Arocena, 2000). Elevated ethylene levels in compacted soils can trigger stress responses that further restrict root development, thereby compromising plant growth and yield (Lin et al., 2010).

Ethylene, a gaseous phytohormone synthesized from methionine in the presence of oxygen, is integral to plant stress responses. It is produced in most plant tissues and serves as a key regulator of various physiological processes, particularly under stress conditions (Lin et al., 2010). Ethylene Response Factors (ERFs) are central to ethylene’s role in stress response, acting as regulatory hubs that interact with other stress-related signals, such as abscisic acid and (ABA) and jasmonate (Müller and Munné-Bosch, 2015). These interactions are critical in modulating plant responses to diverse abiotic stresses, including salinity, drought, low temperature, heat, and variations in light availability. The ethylene signaling pathway is a complex network involving several key components, including receptors, *EIN2*, and transcription factors like *EIN3* (*Ethylene insensitive 3*)*/EIL1 (Ethylene insensitive-Like1).* These components activate ERFs, which, in turn, regulate the expression of genes involved in stress and downstream hormone signaling pathway. Notably, rice mutant lines with alterations in EIN3/EIL1 maintain normal primary root growth even under compacted soil conditions, similar to wild-type plants in non-compacted soils (Pandey et al., 2021). This resilience is likely due to the disrupted ethylene signaling in these mutants, which prevents the typical ethylene-induced inhibition of root elongation in response to soil compaction.

The regulation of root growth and development in rice is a complex process influenced by multiple phytohormones, including auxin, ABA, cytokinin, and ethylene. These hormones are involved in various aspects of root growth and development, from biosynthesis to signal transduction, and play a crucial role in how plants respond and acclimate to environmental stresses (Dello Ioio et al., 2007; De Smet and Jürgens, 2007; McSteen, 2010). Ethylene, in particular, interacts with auxin by modulating its biosynthesis and transport, highlighting its role as a mediator in the regulation of root development (Le et al., 2001). Given the critical role of ethylene in plant stress response, manipulating its pathway offers a promising strategy for improving crop production, especially under adverse conditions. For instance, in maize, mutations in the ACC synthase gene, a key enzyme in ethylene biosynthesis, have been shown to delay leaf senescence and maintain photosynthesis during drought, leading to increased grain yield under stress (Habben et al., 2014). However, breeding for ethylene-responsive traits in crops remains challenging, particularly in non-GMO contexts. Nevertheless, utilizing diverse plant collections and innovative screening protocols can provide valuable genetic resources for breeding programs aimed at enhancing tolerance to unfavorable environments. In rice, developing ethylene-insensitive varieties could be a potential strategy to improve growth and production, particularly in compacted soils where water and nutrient extraction are critical for survival. This approach is especially relevant for countries like Thailand, where traditional breeding methods are preferred over genetic modification. By leveraging the genetic diversity found in local or landrace rice accessions, which often contain adaptive traits to harsh environments, breeders can identify and incorporate valuable alleles into commercial cultivars.

In this study, we aimed to investigate the root length response of 220 rice accessions under controlled and ethylene-treated conditions. By employing a genome-wide association study (GWAS), we sought to identify the genomic regions and key genes regulating root length and ethylene sensitivity in rice. The findings from this study are expected to contribute to the development of new rice varieties with enhanced root penetration ability and improved adaptation to soil compaction and other abiotic stresses.

## 2 Materials and methods

### 2.1 Plant materials and phenotypic screening experiment

A rice diversity panel consisting of 220 rice accessions, including Thai landraces, Thai improved rice varieties, and rice varieties from other countries, were used to represent the diverse geographical regions and rice ecosystems such as rainfed lowland, lowland, upland, deepwater and irrigated system which are found in Thailand (**Supplementary Table S1**). All rice accessions were evaluated ethylene sensitivity using an ethylene chamber to observe root characteristics in response to ethylene. A total of 10-20 seeds per accession were used to screen the ethylene sensitivity. Seeds were first sterilized with 10% sodium hypochlorite and rinsed with distilled water. The seeds were pre-germinated by soaking with distilled water at 30°C in the dark for 2 days then placed on stainless net, each accession was separated with a well. The samples were then put in an air-tight plastic box (27.5 x 39.5 x 25 cm) with a fitted net. The water (200 ml) was added into the box to provide enough moisture and humidity to allow the seedlings to grow in a rice growth room (25°C). The experiments were set into two conditions, ethylene treatment and control. The experimental design was a complete randomized design (CRD) with three replications. For ethylene treatment, the ethylene gas was injected into the plastic box by a calibrated syringe to get 20 ppm concentration. The root length phenotype was observed at 2 days after ethylene treatment following the modified protocols (Ma et al., 2013 and Pandey et al., 2021). For ethephon treatment, the rice seeds were sterilized with 10% sodium hypochlorite for 10 minutes, then rinsed with deionized (DI) water until clean. Next, they were transferred to plates with 20 mL of DI water and incubated in the dark at 30°C for 2 days. After germination, seedlings were placed on stainless steel mesh channels in two chambers: one with 3 L of DI water (control) and one with 100 µM ethephon in 3 L of DI water (treatment). Seedlings were treated for 48 hours, and root lengths were measured using ImageJ software to calculate root length reduction. The percentage of root length reduction is calculated by subtracting the root length in ethylene from the root length in control and multiplied by 100, then divided by root length in control. R version 4.4.1 (R Core Team, 2024) was used to perform data distribution and statistical analysis with R packages in Rstudio (Wickham, 2016; RStudio Team, 2020). 

### 2.2 Population study and genome wide association study analysis

A total of 2,340,775 SNPs with a minor allele frequency (MAF) greater than 5%, missing data less than 10%, and pruned with a 1-kb window were used for population studies and GWAS (**Supplementary Figure S1**). These SNPs were derived from a whole-genome resequencing project conducted by the National Center for Genetic Engineering and Biotechnology, Thailand (Theerawitaya et al., 2022) using Nipponbare IRGSP 1.0 as the reference rice genome. A SNP density heatmap was generated using the CMplot package in RStudio (Yang et al., 2022). Population structure and individual relatedness were determined through principal component analysis (PCA) and kinship analysis using the GAPIT software (Wang and Zhang, 2021). GWAS analysis was performed using the FarmCPU model in GAPIT, with significant SNP associations defined by a uniform threshold of -log_10_(p-value) > 6.

### 2.3 Linkage disequilibrium decay, haplotype analysis and candidate gene identification

Genome-wide LD decay was estimated by PopLDdecay (Zhang et al., 2019) and the LD structure and haplotypes within the QTL region were inferred using LDBlockshow (Zhang et al., 2019). The identification of genes located within the QTL region was based on the annotations from RAP-DB (https://rapdb.dna.affrc.go.jp) (Kawahara et al., 2013; Sakai et al., 2013). The effects of SNP variation in the genes were identified using the Variant Effect Predictor (VEP - https://asia.ensembl.org/info/docs/tools/vep/index.html). The gene-based haplotyped were analyzed using GeneHapR (Zhang et al., 2023) based on the SNPs with a moderate or high effect present in the genes. Candidate genes were identified by conducting an analysis of variance (ANOVA) to test for significant differences between the major haplotypes (each consisting of at least 7 samples) within the QTL.

## 3 Results

### 3.1 Phenotypic variation in root length reduction percentage under ethylene gas exposure in a panel of 220 rice accessions

**Figure 1:**
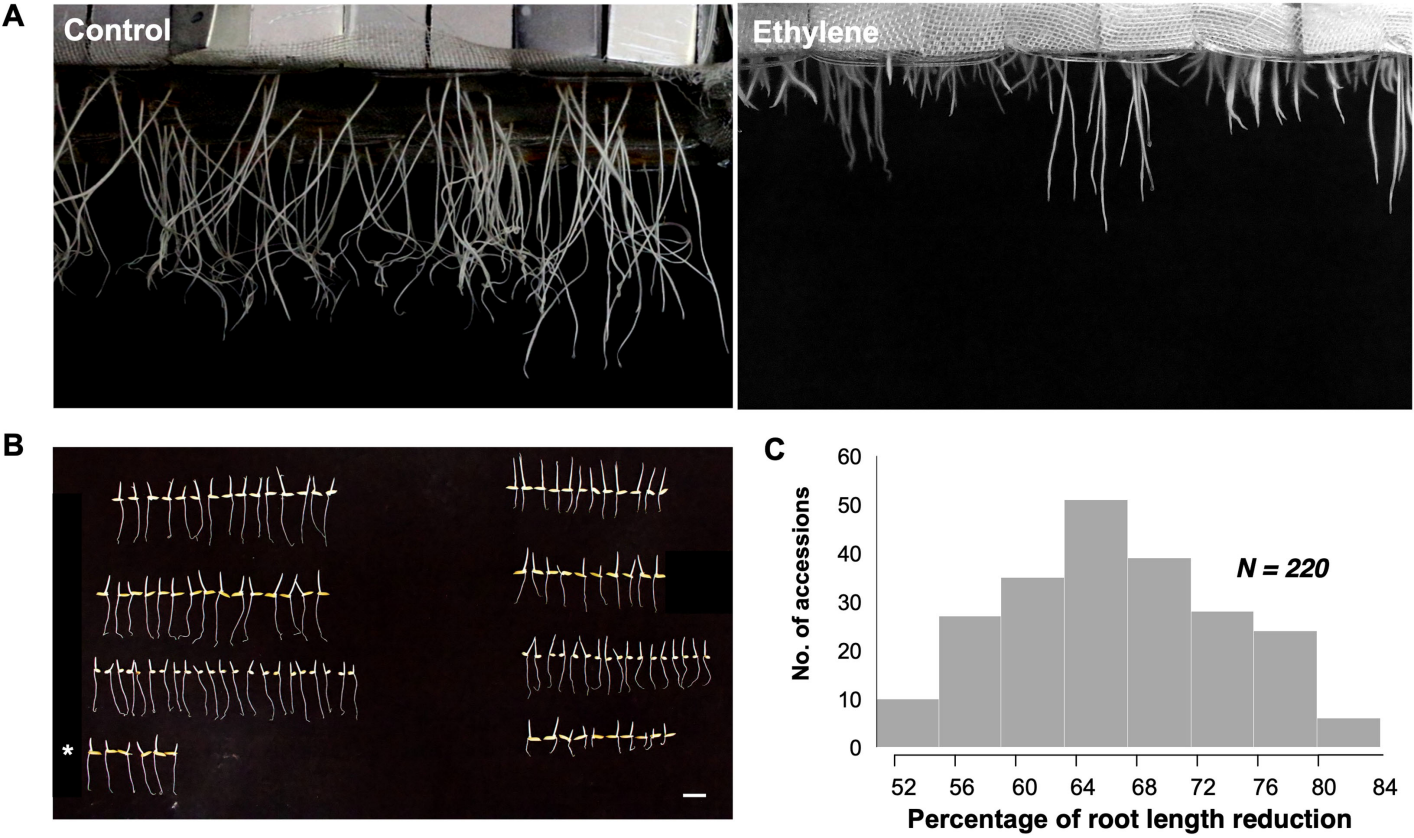
**(A)** Root length reduction under control and ethylene treatments, showing contrasting phenotypes of root length among different accessions. **(B)** Comparison of root length between the two treatments for selected accessions, with KDML105 as the reference check (denoted by *). Left: control; right: ethylene treatment. Scale bar: 2 cm. **(C)** Distribution of data showing the percentage of root length reduction across accessions.

Root responsiveness to ethylene was assessed by calculating the percentage of reduction in root length (%RLR) for each rice accession by comparing the root lengths under ethylene treatment (20 ppm) with control conditions (**Figure 1A**). KDML105 served as a sensitive control to validate ethylene response and ensure reproducibility (**Figure 1B**). The %RLR in this diversity panel ranged from 52.06% to 82.98%, with an average of 67.76% (**Figure 1A**). Rice accessions with a %RLR of 60.67% or lower were classified as low ethylene sensitivity, while those with a %RLR of 74.9% or higher were considered highly sensitive. Naew Ubon 2 exhibited the lowest %RLR (52.06%), and Phitsanulok 80 had the highest %RLR (82.98%). The %RLR distribution across the panel displayed a slightly skew towards low values, forming a continuous curve (**Figure 1C**). The RLR phenotype in rice accessions with low and high sensitivity was confirmed using an ethephon solution treatment (**Supplementary Figure S2**).

### 3.2 Population study and linkage disequilibrium analysis

**Figure 2:**
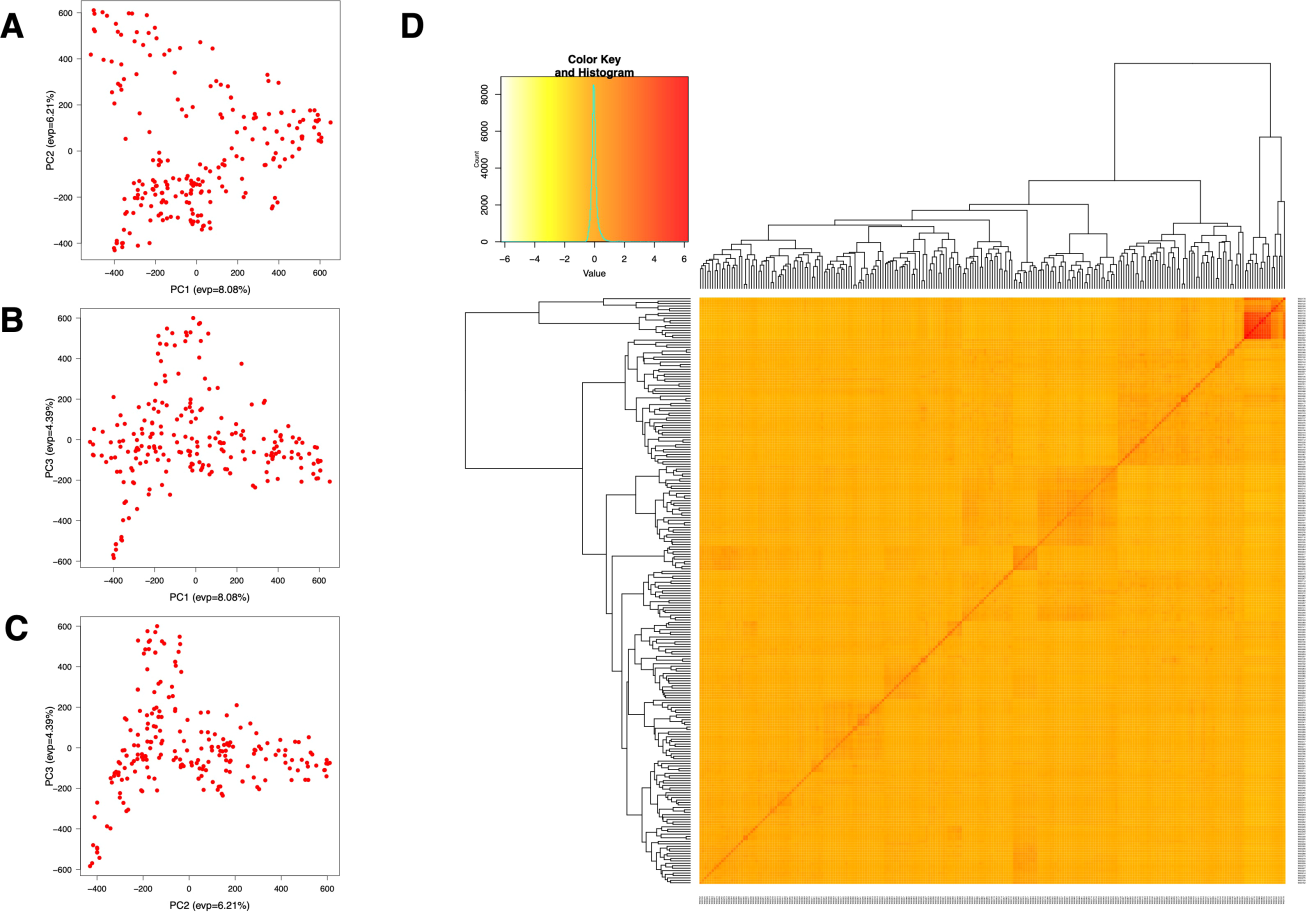
Population structure for GWAS analysis of 220 rice accessions. **(A–C)** Variation captured by the first three principal components (PCs): **(A)** PC1 vs. PC2, **(B)** PC1 vs. PC3, and **(C)** PC2 vs. PC3. **(D)** Kinship matrix of the 220 individuals, presented as a heatmap.

**Figure 3:**
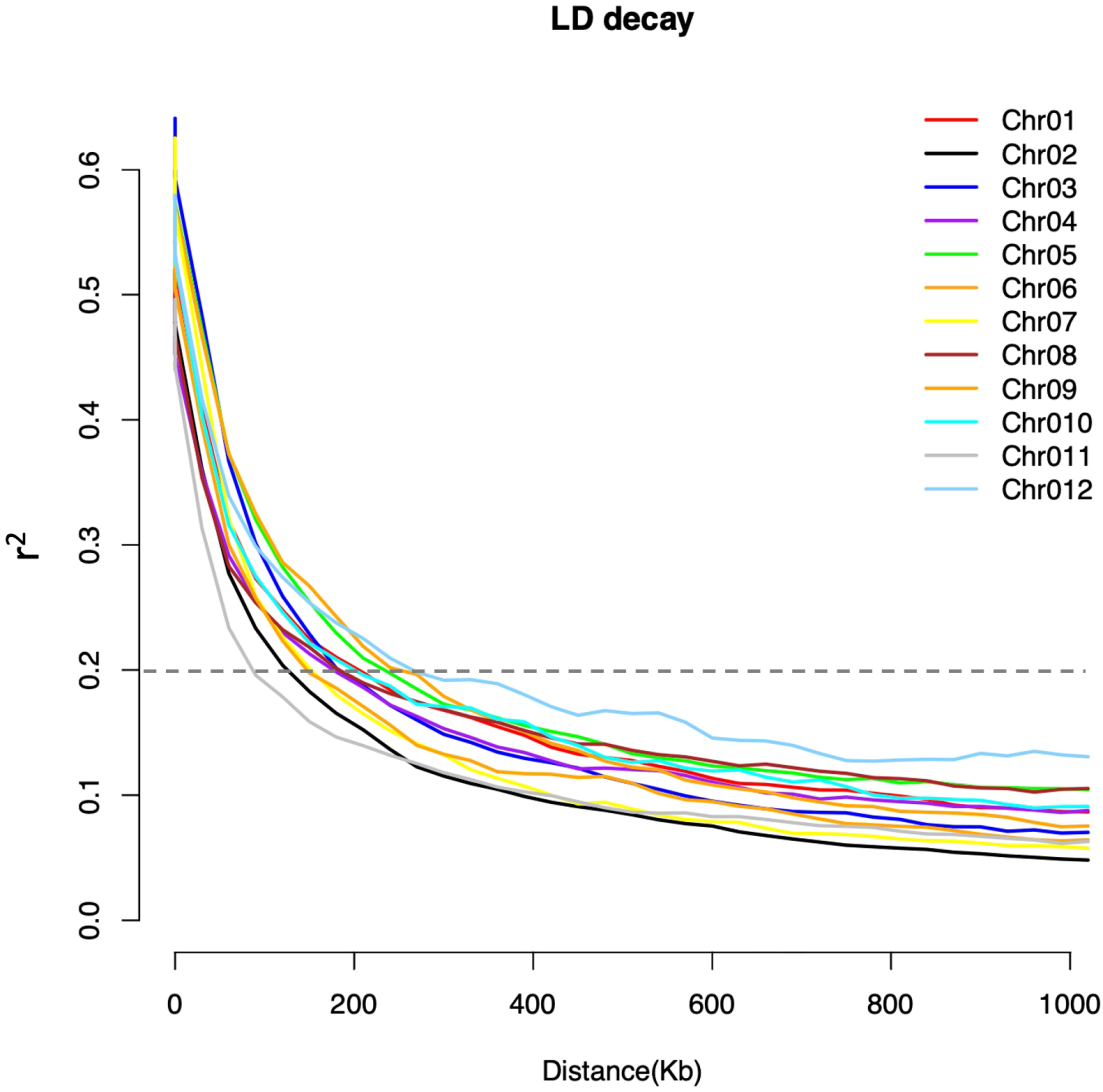
Chromosome-wide linkage disequilibrium (LD) decay estimated from SNP genotypes of 220 rice accessions. Each line represents the smoothed r^2^ values for all marker pairs on each chromosome as a function of the distance between marker pairs. The LD decay threshold (r^2^ = 0.2) is indicated by the dotted line.

The population structure and cryptic relationship among the 220 rice accessions were analyzed using Principal Component Analysis (PCA) and a kinship matrix (**Figure 2**). All rice accessions in this study belong to the indica ecotype (**Supplementary Table S1**). The PCA and VanRaden kinship matrix results indicated the absence of distinct subpopulations within the diversity panel. The kinship coefficient is in the range of 0 to 0.5 which is often used for relatedness in GWAS. To estimate genome-wide linkage disequilibrium (LD) decay, we calculated the pairwise LD index (r^2^) and plotted the LD decay across the rice diversity panel. The average LD decay for the entire genome of the 220 rice accessions was approximately 190 kb (r^2^ < 0.2). Chromosome-wise, the shortest LD decay distance was around 100 kb on chromosome 11, while the longest was approximately 280 kb on chromosome 12 (**Figure 3**).

### 3.3 Genome-wide association analysis and candidate gene identification

**Figure 4:**
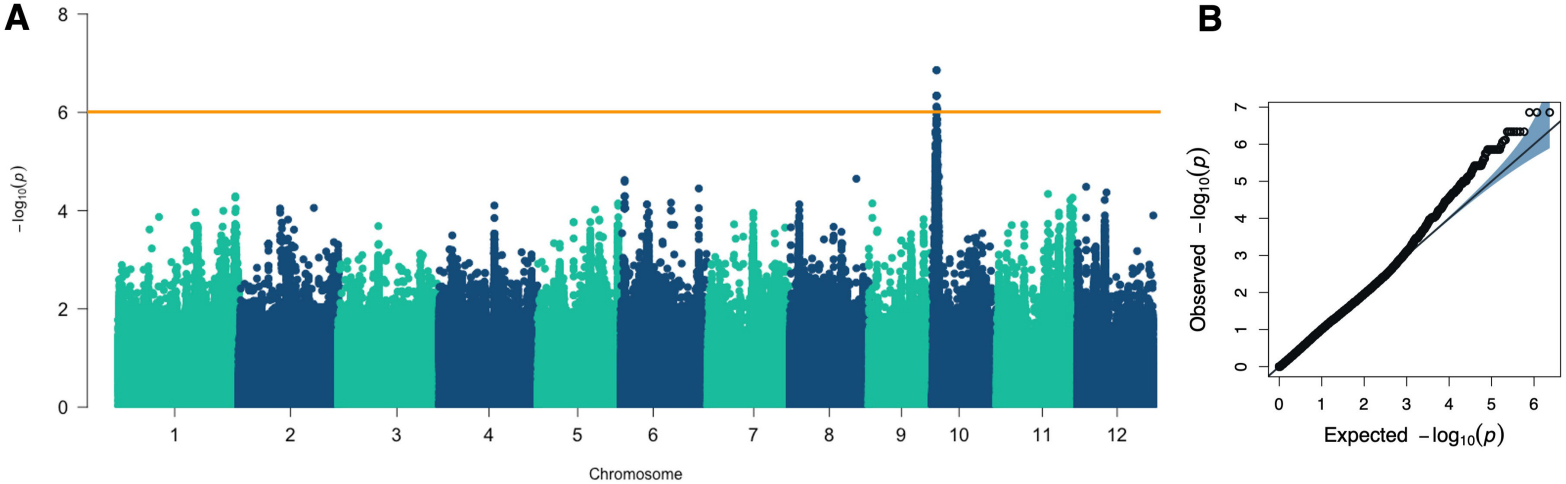
GWAS results for the percentage of root length reduction. **(A)** Manhattan plot from the FarmCPU model displaying the association signals across the genome. **(B)** Quantile-Quantile (Q-Q) plot comparing the distribution of observed versus expected p-values from the GWAS analysis.

To identify genomic regions associated with root responsiveness to ethylene, we conducted a genome-wide association study (GWAS) on 220 rice accessions, utilizing 2,340,775 SNP as genotype data. All SNPs had a minor allele frequency (MAF) greater than 0.05. We employed the FarmCPU model, incorporating the first three principal components (PCs) and the kinship matrix, to detect quantitative trait loci (QTLs). As a result, a quantitative trait nucleotide (QTN) was detected with the highest significance (-log10 p-value of 6.86) on chromosome 10 at position 1,562,158 bp (**Figure 4**). Given the average linkage disequilibrium (LD) decay on chromosome 10, approximately 200 kb (r^2^ = 0.2), we defined the region between 1.36 and 1.76 Mb as the QTL region (named *qRLR10*) associated with root responsiveness to ethylene (**Table 1**).

**Table 1:** The QTLs for percentage of root length reduction based on significant SNPs.

QTL	Chromosome	QTL region (Mb)	Lead SNP	P-value	MAF
*qRLR10*	10	1.36 – 1.76	1562158	1.39 × 10-07	0.44

We then identified candidate genes within the *qRLR10* region by analyzing genes annotated within the LD block containing the lead SNP. A total of 20 genes with annotated functions were identified (**Table 2**). Among these, several genes are related to root development and plant hormone responses, such as *Os10g0122600* (nodulin domain-containing protein, *OsSNDP1*), which is associates with root hair elongation (Huang et al., 2013). In addition, 12 genes encode F-box domain-containing proteins, including *Os10g0123200, Os10g0124500 (OsFbox507, OsFBX343), Os10g0124700 (OsFbox508, OsFBX344), Os10g0125300, Os10g0126000 (OsFbox510, OsFBX346), Os10g0126500 (OsFbox511, OsFBX347), Os10g0126600 (OsFbox512, OsFBX348), Os10g0126700 (OsFbox513, OsFBX346), Os10g0126800 (OsFbox514, OsFBX350), Os10g0127000 (OsFbox515, OsFBX351), Os10g0127900 (OsFbox516, OsFBX352)*, and *Os10g0128200* (*OsFbox517, OsFBX353*). Furthermore, genes encoding CCR4-associated factor 1 (*CAF1*) proteins, *Os10g012390*0 (*OsCAF1-14*) and *Os10g0124200* (*OsCAF1-15*), which are important enzymes for catalysis of mRNA de-adenylation in eukaryotes, were also identified. CAF1 proteins have been reported to response to abiotic stresses in plants. A search of Rice Expression Profile Database (http://ricexpro.dna.affrc.go.jp/) showed that 16 gene expression profiles are in database, and they have different expression patterns, referring their different biological functions. Particularly, *Os10g0122000*, *Os10g0122600*, *Os10g0124700*, *Os10g0125700*, and *Os10g0126000* are expressed in various rice organs throughout rice development, including high expression in root. According to root gene expression profile, *Os10g0122000* is highly expressed in maturation zone, *Os10g0124700* is highly expressed in division zone, *Os10g0122600*, *Os10g0125700*, and *Os10g0126000* are expressed in elongation zone, and maturation I.

**Table 2:** The 20 associated genes within the QTL region containing nonsynonymous SNPs causing missense and nonsense effect.

QTL	Chr.	Start	Stop	Locus ID	Description
*qRLR10*	10	1405692	1407206	*Os10g0122000*	UDP-glucuronosyl/UDP-glucosyltransferase family protein. (Os10t0122000-00)
		1431142	1432407	*Os10g0122300*	Transferase domain containing protein. (Os10t0122300-00)
		1447351	1451372	*Os10g0122600*	Sec14 and nodulin domain-containing protein, Root hair elongation (Os10t0122600-01)
		1479530	1481991	*Os10g0123200*	Cyclin-like F-box domain containing protein. (Os10t0123200-01)
		1525512	1526367	*Os10g0123900*	Similar to CAF1 family ribonuclease containing protein. (Os10t0123900-00)
		1530563	1532176	*Os10g0124000*	Ribosomal protein L7A/RS6 family domain containing protein. (Os10t0124000-01)
		1539462	1540349	*Os10g0124200*	Similar to CAF1 family ribonuclease containing protein. (Os10t0124200-00)
		1556778	1559803	*Os10g0124500*	Cyclin-like F-box domain containing protein. (Os10t0124500-01)
		1562608	1565335	*Os10g0124700*	Cyclin-like F-box domain containing protein. (Os10t0124700-01)
		1622222	1635958	*Os10g0125300*	F-box domain, cyclin-like domain containing protein. (Os10t0125300-01);F-box protein, Tapetum cell development, Pollen formation, Control of anther development (Os10t0125300-02)
		1642565	1646770	*Os10g0125700*	NB-ARC domain containing protein. (Os10t0125700-00)
		1656688	1659249	*Os10g0126000*	Similar to F-box domain containing protein, expressed. (Os10t0126000-01)
		1681092	1684225	*Os10g0126500*	Cyclin-like F-box domain containing protein. (Os10t0126500-01)
		1688331	1691224	*Os10g0126600*	Cyclin-like F-box domain containing protein. (Os10t0126600-01)
		1694920	1697854	*Os10g0126700*	Similar to F-box domain containing protein, expressed. (Os10t0126700-01)
		1700332	1702953	*Os10g0126800*	Cyclin-like F-box domain containing protein. (Os10t0126800-01)
		1711433	1713322	*Os10g0127000*	Similar to F-box domain containing protein, expressed. (Os10t0127000-01)
		1726495	1727783	*Os10g0127350*	Glycosyl transferase, family 14 domain containing protein. (Os10t0127350-00)
		1743579	1752159	*Os10g0127900*	Similar to F-box domain containing protein, expressed. (Os10t0127900-01; Regulation of glucose-induced delay of seed germination (Os10t0127900-03)
		1755462	1757886	*Os10g0128200*	Similar to F-box domain containing protein, expressed. (Os10t0128200-01)

### 3.4 Haplotype analysis

**Figure 5:**
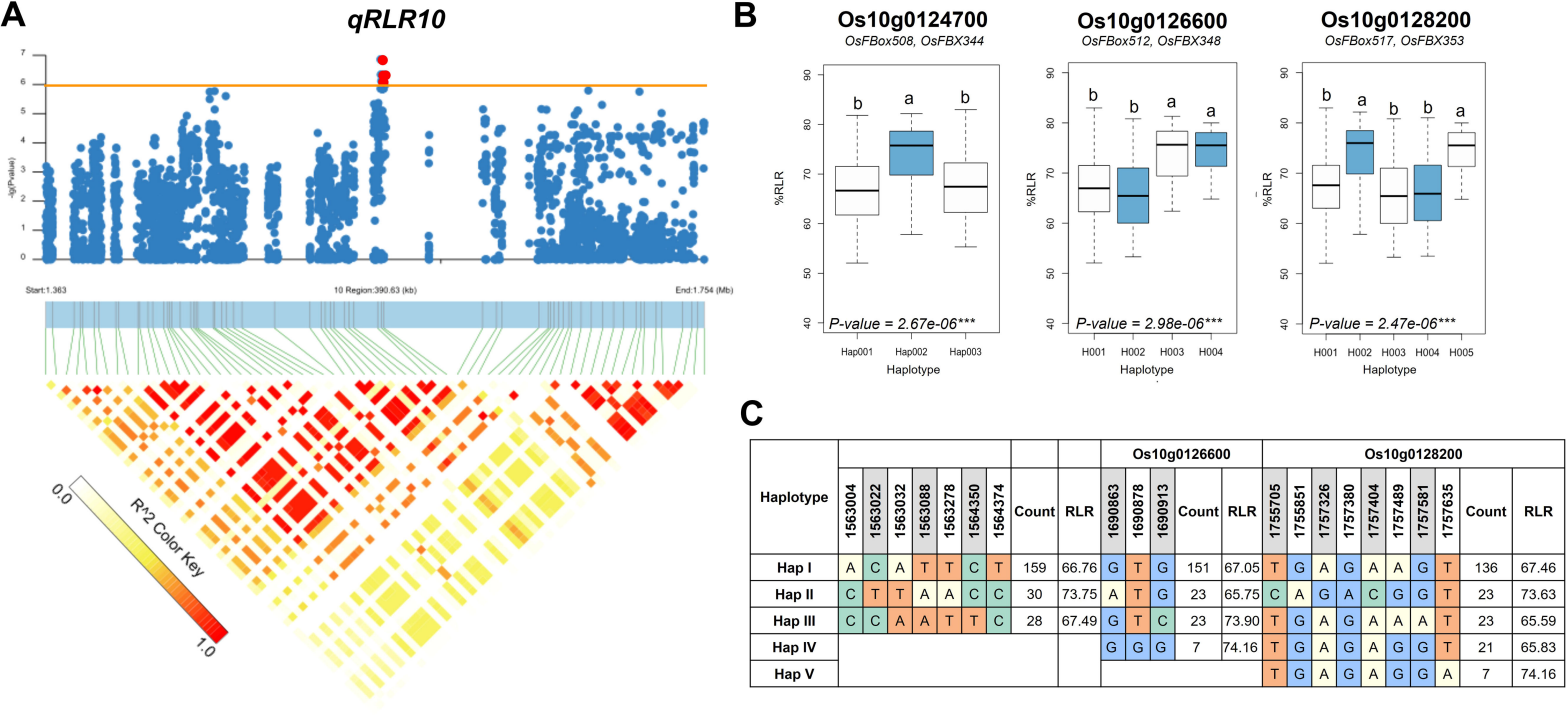
Associated regions and haplotype analysis of *qRLR10*. **(A)** Regional Manhattan plots and LD heatmap across the 390.6-kb region surrounding the significant SNP. Red dots above the red line indicate significant SNPs. Triangles in the LD heatmap represent haploblocks with varying R^2^ values. **(B)** Boxplots displaying the distribution of root length reduction (RLR) across haplotype groups of *Os10g0124700, Os10g0126600*, and *Os10g0128200*. The middle line in each boxplot represents the median. Different letters above each haplotype group show significant difference at p ≤ 0.05. **(C)** Haplotype analysis of the three genes in the region.

To prioritize potential candidate genes, we performed a gene-based haplotype analysis on each of the 20 genes to identify those with haplotypes associated with variations in root length reduction (RLR) within the diversity panel. The analysis focused on SNPs with moderate to high effects, such as nonsynonymous and nonsense SNPs, within each gene. Among the 20 genes analyzed, three—*Os10g0124700*, *Os10g0126600*, and *Os10g0128200*—were found to contain haplotypes significantly associated with the phenotype differences. For *Os10g0124700*, three distinct major haplotypes were identified. Haplotype H002 (n = 30) was significantly associated with a higher %RLR (73.75), whereas haplotypes H001 (n = 159) and H003 (n = 28) were associated with lower %RLR values (66.76 and 67.49, respectively: **Figure 5**). In the case of Os10g0126600, four major haplotypes were found. Haplotypes H001 (n = 151) and H002 (n = 23) were associated with lower %RLR values (67.05 and 65.75, respectively), while the haplotypes H003 (n = 23) and H004 (n = 7) were associated with higher %RLR values (73.90 and 74.16, respectively). For *Os10g0128200*, five major haplotypes were identified. Among these, the haplotypes H002 (n = 23) and H005 (n = 7) were associated with higher %RLR values (73.63 and 74.16, respectively), whereas haplotypes H001 (n = 136), H003 (n = 23) and H004 (n = 21) were associated with lower %RLR values (67.46, 65.59 and 65.83, respectively).

## 4 Discussion

Root plays a vital role in rice growth and performance under both normal and stress conditions, serving as the primary interface between the plant and its environment. The growth, development, and adaptive responses of rice are regulated by a complex network of internal and external factors, including phytohormones such as ethylene, auxin and cytokinin. The hormones can act synergistically or antagonistically to modulate plant growth, particularly under stress conditions (He et al., 2005; Ueda et al., 2020; Yamauchi et al., 2020). Ethylene, often referred to as the stress hormones, is a key mediator in plant adaptive responses, interacting with other hormones to regulate growth under adverse conditions (Huang et al., 2020; Ma et al., 2013; Yang et al., 2015). Roots are particularly sensitive to changes in ethylene concentration, which can lead to reduced root length and increased radial cell expansion (Vaseva et al., 2018). Additionally, ethylene has been shown to influence root hair growth and development (Feng et al., 2017) and plays a role in the plant’s response to soil compaction by reducing root length and increasing root thickness in rice, a process mediated by abscisic acid (ABA) and auxin (Huang et al., 2022). Transgenic rice plants overproducing ethylene exhibit lower 1000-grain weight and faster leaf senescence compared to those with reduced ethylene production, indicating the significant impact of ethylene on overall plant development and yield (Ma et al., 2013). In the ethylene signaling pathway, the ethylene gas triggers the translocation of *CONSTITUTIVE TRIPLE RESPONSE1 (CTR1)* from the endoplasmic reticulum to the nucleus. This process involves the phosphorylation of *ETHYLENE-INSENSITIVE2 (EIN2)* by CTR1, which inhibits EIN2 from signaling in the absence of ethylene (Ju et al., 2012; Jun et al., 2004). In Arabidopsis, plants with enhanced nuclear-localized CTR1 have shown to improve tolerance to drought and salinity stress (Park et al., 2023). These findings suggest that manipulating ethylene sensitivity could be a valuable strategy for improving crop resilience under unfavorable environmental conditions, such as those exacerbated by climate change, while maintaining productivity.

In rice, ethylene’s role in root response to soil compaction has been further elucidated through studies involving rice mutants with varying levels of ethylene sensitivity. For instance, ethylene-insensitive mutants exhibited significantly longer root than those with high ethylene sensitivity under soil compaction, highlighting the genetic basis of ethylene responsiveness (Pandey et al., 2021). In this study, 220 rice accessions were collected from many areas in Thailand in order to have a diverse genetic source to study GWAS. According to Kinship and PCA analysis, our germplasm showed the low levels of relatedness within the rice indica panel (Aesomnuk et al., 2021). Thus, this diversity panel can be used in GWAS analysis. Importantly, the higher genetic diversity of germplasm, the more genetic variation in target trait, leading to successfully identify the crucial gene underlying the target trait (Quero et al., 2018). This will help plant breeder to enhance breeding programs to genetically improve target traits (Mourad et al., 2020). Our genome-wide association study (GWAS) using 220 indica rice accessions identified a QTL, *qRLR10*, associated with difference in root length reduction (RLR) in response to ethylene. Within this QTL region, 20 candidate genes were identified. The subsequent gene-based haplotype analysis revealed that specific haplotypes within three of these genes—*Os10g0124700*, *Os10g0126600*, and *Os10g0128200*—are significantly associated with variations in RLR. This association underscores the potential functional importance of these genes in the regulation of ethylene responsiveness.

The gene *Os10g0124700* (*OsFbox508, OsFBX344*), which encodes an F-box protein, showed the highest expression in rice roots before the flowering stage according to the strand-specific RNA-Seq database in RAP-DB (Wang et al., 2015). F-box proteins are components of the SCF (SKP1-CUL1-F-box protein) complex, which functions as an E3 ubiquitin ligase, tagging target proteins for degradation. This pathway is crucial for regulating various physiological processes, including growth, development, and stress responses (Gagne et al., 2002; Lechner et al., 2006; Dreher and Callis, 2007; Zhang et al., 2008; Guo et al., 2018; Xu et al., 2021). F-box proteins are likely involved in the fine-tuning of ethylene responses by regulation the stability of key component signaling pathway. For example, in Arabidopsis, the F-box protein EBF1/EBF2 targets the ethylene-responsive transcription factor EIN3 for degradation, thus modulating the ethylene response. Given the association of *Os10g012470*0 with variations in RLR, it is plausible that this gene plays a similar role in rice. *Os10g0126600* (*OsFbox512, OsFBX348*) is another F-box domain-containing protein, which has been linked to drought resistance in Shanlan upland rice through its involvement with long non-coding RNA (lncRNA) (Wang et al., 2015). F-box proteins are known to integrate with signaling pathways, including those mediated by phytohormones like ethylene, auxin, and ABA (Koops et al., 2011; Sasaki et al., 2003; Wang et al., 2009; Xu et al., 2021; Yan et al., 2011). The association of *Os10g126600* with RLR suggests its involvement in the regulation of root architecture in response to ethylene, potentially through interactions with other hormones and stress-related pathways. In drought conditions, ethylene production is often elevated, and the ability of roots to adapt to this stress is critical for plant survival. The role of *Os10g0126600* in drought resistance suggests that it may be crucial for modulating root growth under conditions where ethylene levels are altered, helping the plant to balance growth and survival (Yang et al., 2022). This gene’s involvement in RLR further supports its importance in the adaptive response of rice roots to environmental stresses, likely through the modulation of hormone signaling networks. *Os10g0128200* (*OsFbox517, OsFBX353*) has also been identified as a gene associated with RLR, though its specific role in ethylene signaling and root development is less characterized compared to the other two genes. However, its significant association with root length reduction suggest that it may be involved in key regulatory process that control root growth in response to ethylene.

Although not all identified candidate genes showed significant differences in RLR among haplotypes, they may still play roles in root growth and ethylene response. Evidence suggests crosstalk among plant hormones, with ethylene inhibiting rice root growth in compacted soil via ABA and auxin mechanisms (Huang et al., 2022). For example, *OsSNDP1* (Os10g0122600), involved in root hair elongation, might contribute to root responses under compacted soil conditions (Huang et al., 2013; Kong et al., 2024). *OsFbx352* (Os10g0127900) influences ABA biosynthesis and catabolism, affecting root architecture under stress (Song et al., 2012). *OsCAF1-15* (Os10g0124200) is involved in stress hormone responses, suggesting a role in ethylene signaling under compaction (Liang et al., 2009). Additional functional studies on these genes, as well as research on root growth in compacted soil conditions, will be necessary to clearly understand the roles of these genes in ethylene signaling and root development. Understanding the specific functions of these genes could provide valuable insights into the genetic basis of stress resilience in rice, with potential applications in breeding programs aimed at improving crop performance under adverse environmental conditions.

## 5 Conclusion

In this study, we identified a QTL on chromosome 10, *qRLR10*, associated with root length reduction (RLR) in response to ethylene using GWAS in 220 indica rice accessions. Within this QTL region, a cluster of genes encoding F-box proteins were identified as candidate genes, with three of these F-box genes considered particularly promising. These findings highlight the crucial role of ethylene in root development and stress adaptation, and the identification of these candidate genes lays the groundwork for future research and crop improvement strategies.

## Data Availability

The datasets presented in this study can be found in online repositories. The names of the repository/repositories and accession number(s) can be found in the article/**Supplementary Material.**
