# Supplementary material for: Genome-wide association study identifies key F-box genes linked to ethylene responsiveness and root growth in rice (Oryza sativa L.)
Source: Front Plant Sci. 2024 Dec 18;15:1501533. doi: 10.3389/fpls.2024.1501533 (PMC11688335; doi:10.3389/fpls.2024.1501533)
Supplement: Supplementary file 1 [file DataSheet1.pdf]

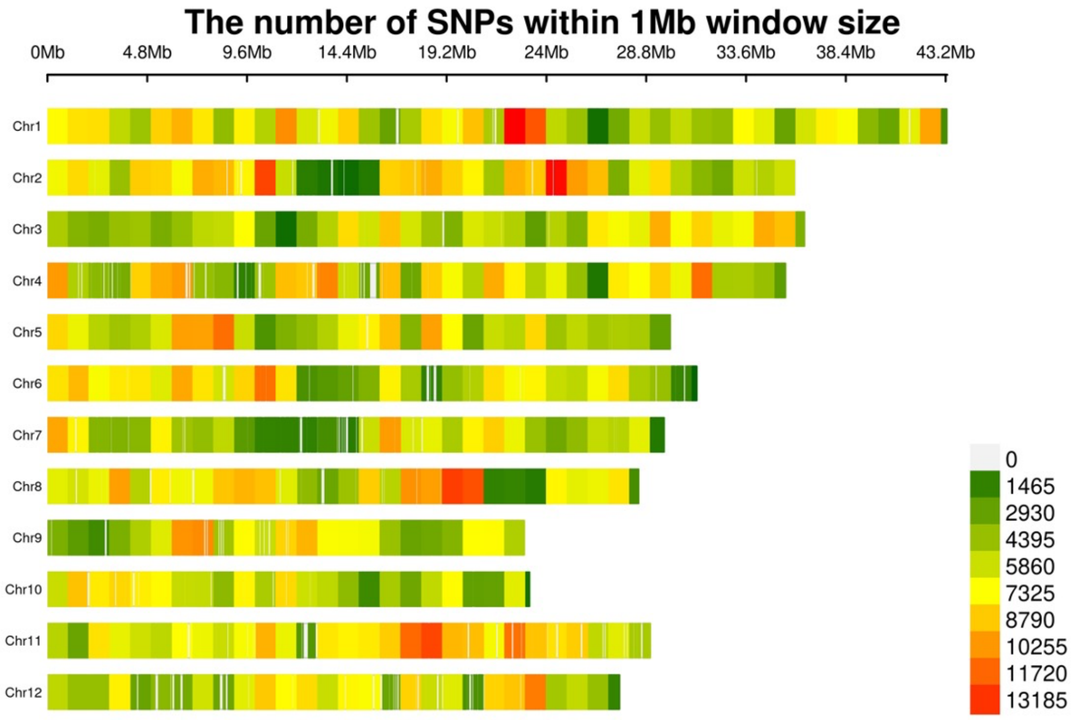

Supplementary Figure 1. Distribution of 1-KB pruned SNPs on 12 rice chromosomes.

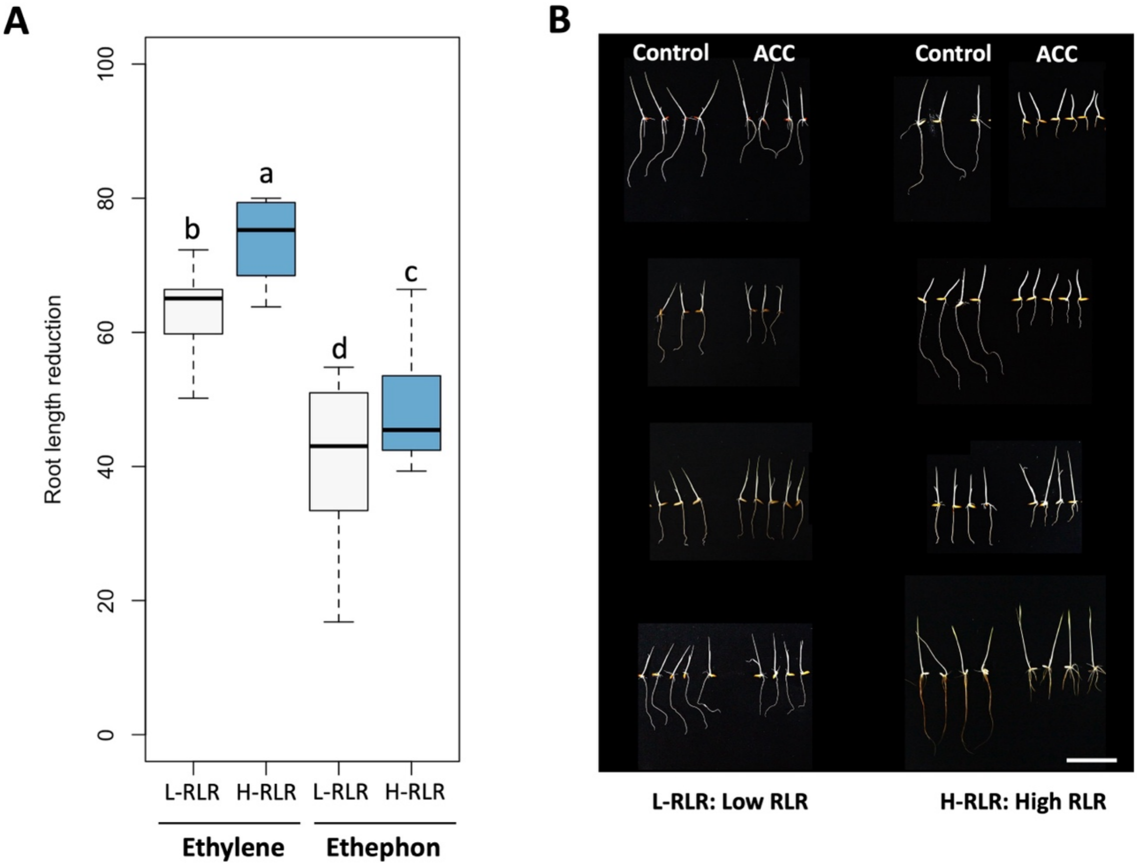

Supplementary Figure 2. (A) Boxplot illustrating the root length reduction (RLR) in rice accessions grouped into two categories—low RLR and high RLR—based on haplotypes in three candidate genes under ethylene treatment and ethephon solution.

(B) Contrasting RLR between the two groups under control and ethephon solution conditions. Scale bar: 5 cm.
