# Supplementary material for: Genome-wide association study identifies key F-box genes linked to ethylene responsiveness and root growth in rice (Oryza sativa L.)
Source: Front Plant Sci. 2024 Dec 18;15:1501533. doi: 10.3389/fpls.2024.1501533 (PMC11688335; doi:10.3389/fpls.2024.1501533)
Supplement: Supplementary file 2 [file DataSheet2.pdf]

Supplementary Material

Supplementary Table 1. List of rice accessions used in this study with code, information and phenotypic data.

Taxa	Name	Ecotype	Type	Origin	%RLR
W00025	Jao Hawm Nin	Indica	Improved variety	Thailand	64.401
W00039	KD	Indica	Unknown	Thailand	76.735
W00053	PSL85063-9-1-2	Indica	Landrace	India	72.465
W00055	Nahng Cha-lawng	Indica	Landrace	Thailand	64.278
W00056	RD69 (Thabthim Chumphae)	Indica	Improved variety	Thailand	58.457
W00059	Khao Nok	Indica	Landrace	Thailand	79.285
W00060	Man Ped 215-33-65	Indica	Landrace	Myanmar	73.454
W00061	BENARA	Indica	Landrace	Unknown	75.989
W00062	Pinkaset 4	Indica	Improved variety	Thailand	65.616
W00063	Khao Jao Hawm Phitsanulok 1	Indica	Improved variety	Thailand	71.477
W00065	Daw Mei		Landrace	Thailand	67.839
W00067	CHEMPAN	Indica	Landrace	Unknown	60.411
W00068	Khao Jao Hawm Supanburi	Indica	Improved variety	Thailand	70.336
W00069	RD51	Indica	Improved variety	Thailand	64.833
W00071	T6-6	Indica	Improved variety	Thailand	62.652
W00072	IR60	Indica	Improved variety	IRRI	67.665
W00074	Khao Gaw Diaw 35	Indica	Landrace	Thailand	76.810
W00075	Surin1	Indica	Improved variety	Thailand	70.705
W00076	CSSL-Chr1 (11)	Indica	Improved variety	Thailand	71.064
W00078	MeeKauk	Indica	Landrace	Thailand	63.240

W00079	HomMali841	Indica	Improved variety	Thailand	70.541
W00080	Khao Jao Hawm Khlong Luang 1	Indica	Improved variety	Thailand	73.037
W00081	RD33	Indica	Improved variety	Thailand	78.056
W00084	Khao Bahn Pong 132	Indica	Landrace	Thailand	53.466
W00086	Khem Tawng Phatthalung	Indica	Landrace	Thailand	63.503
W00087	Supan Buri 3	Indica	Improved variety	Thailand	68.985
W00089	Plai Ngahm Prachinburi	Indica	Landrace	Thailand	69.841
W00090	IR58	Indica	Improved variety	IRRI	72.311
W00091	CSSL-Chr1 (16)	Indica	Improved variety	Thailand	67.621
W00092	Improved Sinthulette-SalTol	Indica	Landrace	Thailand	59.134
W00093	Thunyasirin	Indica	Improved variety	Thailand	68.388
W00094	RD29 (Chai Nat 80)	Indica	Improved variety	Thailand	66.092
W00096	Phitsanulok 80	Indica	Improved variety	Thailand	82.979
W00097	IR40	Indica	Improved variety	IRRI	59.062
W00101	Hawm Cholasit	Indica	Improved variety	Thailand	64.558
W00102	RD-MAEJO2	Indica	Improved variety	Thailand	59.305
W00103	CNTLR82006-KSR-2-7	Indica	Improved variety	Thailand	68.383
W00104	RD43	Indica	Improved variety	Thailand	79.199
W00105	Dee Ngu Lueam	Indica	Landrace	Thailand	55.309
W00106	Mu9962	Indica	Improved variety	Thailand	62.096
W00107	RD49	Indica	Improved variety	Thailand	66.197

W00108	RD7	Indica	Improved variety	Thailand	81.307
W00109	Supan Buri 2	Indica	Improved variety	Thailand	70.200
W00110	RD41	Indica	Improved variety	Thailand	61.806
W00111	RD4	Indica	Improved variety	Thailand	68.638
W00112	Supan Buri 1	Indica	Improved variety	Thailand	68.334
W00113	RD21	Indica	Improved variety	Thailand	78.412
W00114	Pathum Thani 1	Indica	Improved variety	Thailand	54.957
W00116	1094-61	Indica	Improved variety	Thailand	63.801
W00117	BA7	Indica	Landrace	Unknown	61.669
W00118	Basmati 370	Indica	Improved variety	India	75.521
W00121	FL496	Indica	Improved variety	IRRI	76.096
W00122	FR13A	Indica	Landrace	India	73.257
W00123	HomMali803-1	Indica	Improved variety	Thailand	68.378
W00124	HomMali803-2	Indica	Improved variety	Thailand	81.840
W00125	HomMali821	Indica	Improved variety	Thailand	72.517
W00126	IR1188	Indica	Improved variety	Unknown	77.322
W00127	IR64	Indica	Improved variety	IRRI	69.002
W00128	IR72	Indica	Improved variety	IRRI	64.643
W00131	PK1-PY-BLB-2	Indica	Landrace	Thailand	78.358
W00134	Niaw Ubon1	Indica	Improved variety	Thailand	55.420
W00136	RD14	Indica	Improved variety	Thailand	59.484

W00137	RD23	Indica	Improved variety	Thailand	78.649
W00140	Daw Sahm Deuan	Indica	Landrace	Thailand	70.591
W00141	Nahng Khiaw	Indica	Landrace	Thailand	52.247
W00144	Supan Buri 4	Indica	Improved variety	Thailand	58.747
W00145	Hawm Poo Khiaw	Indica	Landrace	Thailand	70.560
W00146	Hawm Supan	Indica	Improved variety	Thailand	71.216
W00154	Kalubala Vee	Indica	Landrace	Sri Lanka	64.478
W00156	Koo Mueang	Indica	Landrace	Thailand	66.524
W00157	Chaw Ma-kawk	Indica	Landrace	Thailand	65.516
W00158	Dawk Mai	Indica	Landrace	Thailand	69.674
W00159	Ta Pow Lom	Indica	Landrace	Thailand	71.031
W00160	Tah Ban	Indica	Landrace	Thailand	67.380
W00161	Tawng Rahk Sai	Indica	Landrace	Thailand	56.886
W00162	PraTan Bahn Boong	Indica	Landrace	Thailand	62.418
W00164	Mueang Ngah	Indica	Landrace	Lao PDR	59.626
W00165	Rueng Diaw	Indica	Landrace	Thailand	61.073
W00166	Lao Taeg	Indica	Landrace	Thailand	61.567
W00170	DULAR(GS.20018)	Indica	Landrace	Thailand	68.274
W00172	Hom Soo Tabut	Indica	Landrace	Thailand	78.173
W00173	299 Hom Lanna	Indica	Improved variety	Thailand	65.259
W00174	716 54R	Indica	Improved variety	Unknown	69.399
W00175	Abhaya	Indica	Improved variety	Unknown	73.047
W00178	DH103	Indica	Improved variety	Unknown	64.817
W00179	DH212	Indica	Improved variety	Unknown	57.419
W00180	DV85	Indica	Improved variety	Unknown	66.660
W00181	FL530	Indica	Improved variety	Unknown	69.292

W00183	HomMali805	Indica	Improved variety	Thailand	63.119
W00185	IR49830	Indica	Improved variety	IRRI	64.358
W00186	IR53936	Indica	Improved variety	IRRI	66.989
W00187	IR57514	Indica	Improved variety	IRRI	79.525
W00188	IR62266	Indica	Improved variety	IRRI	76.468
W00189	IWS10	Indica	Improved variety	Thailand	77.257
W00190	Jasmine IR57514 (BC4F7)	Indica	Improved variety	Thailand	67.817
W00191	KDML105-PlusIII	Indica	Improved variety	Thailand	61.699
W00192	MNTK 75	Indica	Landrace	India	75.562
W00194	No.51-PSL	Indica	Landrace	India	69.017
W00196	PTB33	Indica	Improved variety	#N/A	59.256
W00197	Rathu heenati	Indica	Improved variety	#N/A	75.526
W00198	TDK1_Sub1	Indica	Improved variety	Lao PDR	59.052
W00199	Gao Rueng 88	Indica	Landrace	Thailand	64.425
W00201	Khiaw Leu	Indica	Landrace	Thailand	64.930
W00204	Chiang Pattalung	Indica	Landrace	Thailand	55.371
W00205	Leb Nok Pattani	Indica	Landrace	Thailand	69.801
W00207	Shian On	Indica	Landrace	Thailand	67.120
W00208	Niaw Khiaw Ngoo (GS.7545)	Indica	Landrace	Thailand	71.580
W00209	Niaw Dam	Indica	Landrace	Thailand	74.552
W00210	Niaw San-pah Tawng	Indica	Improved variety	Thailand	70.417
W00211	Niaw Hawm (GS.4915)	Indica	Landrace	Thailand	76.449
W00212	Niaw Ubon2	Indica	Improved variety	Thailand	52.061
W00213	Muey Nawng 62 M	Indica	Landrace	Thailand	75.838

W00214	Leuang Yai	Indica	Landrace	Thailand	67.085
W00216	Leuang Kha-min	Indica	Landrace	Thailand	70.718
W00217	Luang Pratew 123	Indica	Landrace	Thailand	81.037
W00218	Leuang Plai Lah	Indica	Landrace	Thailand	64.236
W00219	Leuang Hawm	Indica	Landrace	Thailand	53.425
W00220	Phare	Indica	Landrace	Thailand	62.011
W00222	Khai Mod Rin 3	Indica	Landrace	Thailand	74.599
W00224	RD10	Indica	Improved variety	Thailand	79.853
W00225	RD11	Indica	Improved variety	Thailand	67.532
W00227	RD27	Indica	Improved variety	Thailand	64.249
W00228	RD31	Indica	Improved variety	Thailand	74.312
W00229	RD37	Indica	Improved variety	Thailand	76.747
W00231	RD5	Indica	Improved variety	Thailand	64.568
W00232	RD6	Indica	Improved variety	Thailand	73.470
W00233	RD8	Indica	Improved variety	Thailand	65.065
W00235	Gon Gaew (GS.1698)	Indica	Landrace	Thailand	76.021
W00236	Goo Meuang Luang	Indica	Landrace	Thailand	65.237
W00241	Khao Tah Haeng 17	Indica	Landrace	Thailand	82.175
W00242	Khao Niaw Nah 432	Indica	Landrace	Thailand	69.598
W00243	Khao Bun Mah	Indica	Landrace	Thailand	80.825
W00244	Khao Pahn Luang	Indica	Landrace	Thailand	60.999
W00246	Khi Tom Pan	Indica	Landrace	Thailand	60.556
W00247	Ngah Chang	Indica	Landrace	Thailand	72.178
W00249	Chai Nat 1	Indica	Improved variety	Thailand	78.262
W00250	Chai Nat 2	Indica	Improved variety	Thailand	67.543

W00251	Chum Phae 60	Indica	Improved variety	Thailand	58.957
W00253	Daw Khao GS.5584	Indica	Landrace	Thailand	56.624
W00255	Ta Pow Gaew 161	Indica	Landrace	Thailand	63.529
W00256	Tab Mei Dam	Indica	Landrace	Thailand	62.550
W00257	Nahng Mon S-4	Indica	Landrace	Thailand	76.618
W00260	Bak Muay	Indica	Landrace	Thailand	67.062
W00261	Bang Taen	Indica	Improved variety	Thailand	80.006
W00262	Beu Pah Toh	Indica	Landrace	Thailand	62.364
W00264	Prachin Buri1	Indica	Improved variety	Thailand	77.987
W00265	Prachin Buri2	Indica	Improved variety	Thailand	77.116
W00266	Plong Aew	Indica	Landrace	Thailand	65.847
W00267	Pinkaset 3	Indica	Improved variety	Thailand	66.802
W00268	Pin Gaew 56	Indica	Landrace	Thailand	58.131
W00271	Pamah Dam	Indica	Landrace	Thailand	71.577
W00273	Pahn Tawng	Indica	Landrace	Thailand	72.295
W00275	Phitsanulok 60-1	Indica	Landrace	Thailand	77.269
W00277	Mali Daeng	Indica	Improved variety	Thailand	72.575
W00278	Mali Gomen	Indica	Landrace	Thailand	70.386
W00281	Look Leuang	Indica	Landrace	Thailand	77.964
W00282	Look Daeng Pattani	Indica	Landrace	Thailand	65.715
W00283	Sangyod	Indica	Landrace	Thailand	66.943
W00284	Sangyod Phatthalung	Indica	Landrace	Thailand	58.969
W00285	San Pah-tawng 1	Indica	Improved variety	Thailand	66.717
W00286	Sin Lek	Indica	Improved variety	Thailand	68.500
W00287	Supanburi 60	Indica	Improved variety	Thailand	72.259
W00288	Supanburi 90	Indica	Improved variety	Thailand	75.637

W00289	Mahk Nam	Indica	Landrace	Thailand	57.165
W00290	Luang Pratan (GS.6440)	Indica	Landrace	Thailand	63.838
W00291	Hom Khee Kai	Indica	Landrace	Thailand	59.462
W00292	Hawm Jan(GS.3008)	Indica	Landrace	Thailand	66.295
W00293	Hawm Dong	Indica	Landrace	Thailand	69.661
W00294	Hawm Nai Pon	Indica	Landrace	Thailand	69.337
W00295	Hawm Pamah (GS.5333)	Indica	Landrace	Thailand	64.265
W00297	Han Trah 60	Indica	Improved variety	Thailand	79.769
W00298	Hahng Yi 71	Indica	Landrace	Thailand	72.062
W00299	Hah Ruang Bow	Indica	Landrace	Thailand	57.173
W00300	Ayutthaya 1	Indica	Improved variety	Thailand	67.772
W00301	Niaw Hawm (GS.9194)	Indica	Landrace	Thailand	73.193
W00302	Khao Dawk Mali 105	Indica	Landrace	Thailand	66.618
W00303	Khao Tah Haeng	Indica	Landrace	Thailand	57.333
W00304	Pin Gaew	Indica	Landrace	Thailand	60.822
W00305	Pueng Tawng (GS.574)	Indica	Landrace	Thailand	53.274
W00307	Hahng Nahk	Indica	Landrace	Thailand	66.145
W00308	RD53	Indica	Improved variety	Thailand	69.864
W00309	Kon Jud	Indica	Landrace	Thailand	65.438
W00310	Kao Gam	Indica	Landrace	Thailand	65.257
W00312	Jao Khao	Indica	Landrace	Thailand	76.011
W00314	Dawk Khah	Indica	Landrace	Thailand	71.289
W00315	Daw Dawk Mai	Indica	Landrace	Thailand	73.199
W00317	Tom Meuang Luang	Indica	Landrace	Thailand	68.133
W00318	Tah-khiad	Indica	Landrace	Thailand	73.231
W00320	Nah Khawn	Indica	Landrace	Thailand	59.131
W00321	Puang Sung	Indica	Landrace	Thailand	58.130
W00322	Puang Hahng Nahk	Indica	Landrace	Thailand	65.900
W00323	Rahk Haeng	Indica	Landrace	Sri Lanka	54.352
W00324	Hawm Tawng	Indica	Landrace	Thailand	58.687
W00325	Hawm Nahng Naun	Indica	Landrace	Thailand	66.908

W00326	Niaw Mali	Indica	Landrace	Thailand	59.703
W00327	Leuang Kai Lah (GS.5555)	Indica	Landrace	Thailand	60.546
W00328	Leuang Ngahm	Indica	Landrace	Thailand	62.914
W00331	CNT1-Qbph6,12(fromAB)	Indica	Improved variety	Thailand	59.006
W00332	CNTBR82040-259-1-1-1	Indica	Improved variety	Thailand	76.914
W00333	CSSL-Chr8 (106)	Indica	Improved variety	Thailand	58.588
W00334	HomMali802	Indica	Improved variety	Thailand	63.197
W00335	HomMali823	Indica	Improved variety	Thailand	59.966
W00336	IR4563-52-1-3-6	Indica	Improved variety	IRRI	65.251
W00337	KD20	Indica	Improved variety	Thailand	63.401
W00338	KDML105-Bph3-1	Indica	Improved variety	Thailand	62.894
W00339	TDK1_Aroma	Indica	Improved variety	Lao PDR	71.524
W00340	Chiang Dao	Indica	Landrace	Thailand	61.854
W00341	Gaen Jan	Indica	Landrace	Thailand	74.491
W00342	Mae Lahd	Indica	Landrace	Thailand	61.874
W00343	RD13	Indica	Improved variety	Thailand	80.045
W00344	RD35	Indica	Improved variety	Thailand	61.407
W00345	RD47	Indica	Improved variety	Thailand	66.851
W00346	Gon Gaew (GS.6158)	Indica	Landrace	Thailand	57.824
W00347	Khaw Glang	#N/A	Unknown	Unknown	75.800
W00348	Pinkaset 1	Indica	Improved variety	Thailand	73.872
W00350	Sinuan	Indica	Landrace	Thailand	67.286
W00351	Hawm Daeng Noi	Indica	Landrace	Thailand	60.893
W00352	E-pid	Indica	Improved variety	Myanmar	70.311

W00354	Daw Khao GS.12155	Indica	Landrace	Thailand	76.759
W00356	Hawm Pamah (GS.19843)	Indica	Landrace	Thailand	76.522
